# A Rapidly Fatal Case of Low-Dose Methotrexate Toxicity

**DOI:** 10.1155/2018/9056086

**Published:** 2018-06-13

**Authors:** Nasreen Shaikh, Muhammad Sardar, Rishi Raj, Punit Jariwala

**Affiliations:** Department of Internal Medicine, Monmouth Medical Center, 300 Second Avenue, Long Branch, NJ 07740, USA

## Abstract

An 82-year-old female presented with multiple oral ulcers and malena for 1 week. Her laboratory tests revealed pancytopenia and acute renal failure. She had history of rheumatoid arthritis for which she was taking 7.5 mg methotrexate weekly and stage 4 chronic kidney disease from diabetic nephropathy. During the hospital stay, she developed pneumonia and septic shock requiring norepinephrine and vasopressin. She underwent continuous venovenous hemodiafiltration. Leucovorin, filgrastim, and multiple packed red blood cell and platelet transfusions were given. She remained hypotensive and pancytopenic despite all interventions. She died on day 6 of hospital stay from acute hypoxic respiratory failure due to septic shock.

## 1. Introduction

We wanted to report this case to create awareness that methotrexate toxicity can be fatal. Although a low-dose regimen is used in the treatment of rheumatoid arthritis, it can be potentially toxic in the presence of chronic kidney disease especially in the elderly with multiple comorbidities. The American College of Rheumatology guidelines have given recommendations regarding monitoring complete blood count (CBC), liver transaminases, and creatinine and mention that in the presence of comorbidities more frequent monitoring is warranted; however; specific guidelines in the presence of chronic kidney disease are lacking. Methotrexate should be used with utmost caution in the presence of chronic kidney disease.

## 2. Presentation

An 82-year-old female presented to the emergency room with complaints of bleeding oral ulcers and black tarry stools for 1 week. Her past medical history was significant for paroxysmal atrial fibrillation for which she was taking apixaban, rheumatoid arthritis for which she was taking 7.5 mg methotrexate once a week for 6 years, coronary artery disease with left circumflex and right coronary artery stent placement 6 years ago, congestive heart failure with reduced ejection fraction for which she was taking digoxin and furosemide, diabetes mellitus type 2 on repaglinide, hypertension, and stage 4 chronic kidney disease secondary to diabetes and hypertension. At the time of presentation, her vitals were stable. She had multiple bleeding oral ulcers, and digital rectal exam did not show fresh blood but occult blood was positive.

Her complete blood count revealed a hemoglobin of 8.1 g/dL with MCV of 106.8 fL consistent with macrocytic anemia, platelet count of 73 × 10^3^/*µ*L, WBC count of 4.0 × 10^3^/*µ*L with absolute neutrophil count of 500, sodium 147 mEq/L, potassium 6.0 mEq/L, bicarbonate 14 mEq/L, creatinine 7.75 mg/dL, BUN 125 mg/dL, eGFR 5 ml/min/1.73 m^2^, ALT 30 U/L, AST 26 U/L, alkaline phosphatase 56 U/L, digoxin level of 1.3 ng/mL, and methotrexate level of 0.27 *µ*mol/L. 12-lead EKG revealed the first degree AV block, and telemetry showed frequent pauses of 2.5 seconds and escalation to type 2 block at times. Chest X-ray on admission showed cardiomegaly but no pulmonary disease. Patient's advanced directives indicated that she did not want resuscitation.

Her previous lab results were reviewed, and it was noted that the decline in renal function occurred over a span of two weeks. She had renal function tests done 2 weeks prior to the present admission which showed the beginning of worsening renal function as compared to her baseline with a creatinine of 3.58 mg/dL and eGFR of 12 ml/min/1.73 m^2^. She however continued to take Methotrexate.

During day 1 of hospital stay, she had 2 episodes of hematemesis after which hemoglobin and platelets dropped to 7.4 g/dL and 29 × 10^3^/*µ*L, respectively. Her WBC count was 500/mm^3^ and absolute neutrophil count dropped to zero. The patient was given intravenous leucovorin 100 mg every 6 hours and filgrastim 480 mcg subcutaneously once daily, as she was neutropenic. She was given 2 units of packed red blood cell and 1 unit of platelet transfusion. Hemoglobin and platelet levels came up to 8.1 g/dL and 30 × 10^3^/*µ*L, respectively. Darbepoetin 60 mcg subcutaneous was also administered. Intravenous normal saline with 40 mEq bicarbonate was given at 50 cc/hr. Hemodialysis was initiated; however, patient became hypotensive with her MAP ranging from 55 to 60 mmHg. Continuous venovenous hemodiafiltration (CVVHFD) was then started.

On day 2 of hospital stay, she spiked fevers of 101.2F. Chest X-ray was repeated and showed left mid and lower lung field opacity suggestive of consolidation. Blood cultures were immediately sent and IV meropenem 1 g every 12 hours, IV vancomycin 1 gram dosed daily according to trough levels, and IV caspofungin 50 mg every 24 hours were initiated empirically. Blood cultures grew *Klebsiella pneumoniae*. Broad spectrum antibiotics were continued. MTX levels trended down to 0.18 *µ*mol/L. Absolute neutrophil count only came up to 100/mm^3^. Her oxygen saturation was maintained over 90% with the help of high-flow nasal cannula at 40 L/min.

On day 3, she became increasingly hypotensive due to septic shock requiring norepinephrine at 14 mcg/min. Vasopressin was also added at 0.03 units/min. Leucovorin rescue was discontinued as MTX levels were now 0.02 *µ*mol/L. Platelets dropped to 13 × 10^3^/*µ*L, hence 1 unit of platelets were transfused. Despite administering filgrastim, the absolute neutrophil count again dropped to zero with a WBC count of 300/mm^3^. Intravenous hydrocortisone 100 mg every 8 hours was also initiated for septic shock. On day 4, the patient continued to require high-doses of norepinephrine and vasopressin. Repeat blood cultures were negative.

She remained hypotensive requiring increasing amount of norepinephrine. Neutropenia, anemia, and thrombocytopenia persisted. Goals of care were discussed with the family on day 5, and it was decided to stop CVVHFD and withdraw care while initiating comfort measures. The patient expired on day 6 of hospital stay from acute hypoxic respiratory failure due to septic shock.

## 3. Discussion

This is a case of an elderly woman with multiple comorbidities who experienced a fatal decline in her health that was triggered by continuation of low-dose methotrexate therapy for rheumatoid arthritis, which generally is an excellent drug for inflammatory arthritis with a good safety profile, despite worsening renal function. Renal dysfunction led to accumulation of toxic levels of methotrexate resulting in pancytopenia and making the patient susceptible to infections and severe sepsis ultimately causing her demise.

Methotrexate is currently considered the first-line disease-modifying antirheumatic drugs (DMARDs) [1, 2]. It is an antimetabolite that competitively inhibits the conversion of dihydrofolate to tetrahydrofolate by binding to dihydrofolate reductase. Tetrahydrofolate is essential for the synthesis of thymidine and purines required for DNA synthesis. High-dose methotrexate treatment is defined as a dose greater than 500 mg/m^2^ given intravenously and is mostly used in the treatment of various malignancies [3]. Low-dose regimen (5 mg to 25 mg once weekly) has been widely and safely used in the treatment of rheumatoid arthritis, psoriasis, and many other rheumatologic diseases.

The prevalence of rheumatoid arthritis in elderly is increasing over last two decades, and in many countries, the prevalence can be as high as 40% in the elderly patient (older than 65 years) [4]. Many studies have proved its safety in this group of patients. In 1995, Felson et al. did analyses on pooled data from 11 clinical trials comprising of 496 patients, which showed neither age nor renal impairment had any effect on the efficacy of methotrexate; however, its toxicity remained unchanged in elderly but significantly increased in the patient with reduced renal function [5]. In 1996, Bologna et al. studied effects of low-dose methotrexate treatment in 469 patients and concluded that age at initiation of methotrexate treatment did not influence its efficacy or toxicity [6]. This led to the conclusion that reduced renal function in our patient might be responsible for severe toxicity.

As over 90% of methotrexate is excreted and unchanged renally by the mechanism of glomerular filtration, tubular secretion, and tubular reabsorption, its elimination half-life increases and the clearance decreases with the degree of renal impairment [7]. Medication causing decreased renal elimination (aminoglycosides, cyclosporine, nonsteroidal anti-inflammatory agents, sulfonamides, probenecid, salicylates, penicillin, colchicine, cisplatin, and other nephrotoxic drugs) and drugs causing displacement of methotrexate from its protein-binding sites (salicylates, probenecid, sulfonamides, barbiturates, phenytoin, retinoids, sulfonylureas, and tetracyclines) significantly increase its toxicity.

The MATRIX study (Methotrexate and Renal Insufficiency study) conducted in 2004 concluded that serum creatinine alone is not enough to evaluate renal failure in patients taking methotrexate and suggested to follow creatinine clearance measurement [8]. Since then, many guidelines have been published regarding dose adjustments of methotrexate based on renal impairment. University College London Hospital compiled together many references and suggested that methotrexate dosing should be reduced to 65% for creatinine clearance below 60 ml/min and to 50% for creatinine clearance below 45 ml/min. Holding methotrexate all together was suggested if creatinine clearance is under 30 ml/min. The concomitant use of other nephrotoxic drugs as mentioned above is also an important factor in dose adjustment of methotrexate in patients with kidney dysfunction. It is unclear why our patient continued to take methotrexate despite worsening renal function. She was an elderly lady on multiple medications who lived by herself, and it can be speculated that she was not able to care for herself adequately.

Needless to say, patients on methotrexate need regular laboratory tests to monitor their kidney function, liver function, and blood counts. The American College of Rheumatology has guidelines for following patients on methotrexate with routine blood tests consisting of a complete blood count, liver function test, and creatinine. If the patient has been on the medication for more than 6 months, blood tests are required to be done every 12 weeks. However, in the presence of comorbidities or abnormal lab results, more frequent monitoring is warranted [9].

If not adjusted renally, it can lead to adverse events most commonly manifesting in the form of mucositis, hepatotoxicity, nephrotoxicity, and myelosuppression. Most of the toxicity profile has been studied in patients taking high-dose methotrexate for cancers. Few studies have been done on the toxicity of low-dose methotrexate. In a study by Kivity et al. clinical characteristics and risk factors for low-dose methotrexate toxicity were studied in 28 patients. Pancytopenia was the most common manifestation of low-dose methotrexate toxicity seen in 78.5% of the cohort while hepatoxicity in the form of mild elevation of liver enzymes was seen in only 28%. This is in accordance with our patient who presented with pancytopenia with normal liver function tests. They also found that drug level monitoring did not correlate with the degree of pancytopenia and the serum drug levels did not differ in patients who died from toxicity from those who recovered from it [10]. Nonetheless, there are recommendations to trend methotrexate levels until the level is <0.1 *µ*M [11]. In another case study by Calvo-Romero et al., it was found that pancytopenia due to low-dose methotrexate therapy is more likely to occur in the presence of renal impairment [12]. Mori et al. retrospectively analyzed 40 cases of low-dose methotrexate-induced myelosuppression and concluded that myelosuppression can occur abruptly at any time during treatment. They also mentioned that serum albumin levels and folic acid supplementation were the most important risk factors affecting the severity of pancytopenia. Our patient was not taking folic acid supplements and was found to have severe pancytopenia [13].  Table 1 summarizes studies, case series, and case reports regarding low-dose methotrexate toxicity.

Management of methotrexate toxicity is aimed towards giving folinic acid rescue and enhancing methotrexate elimination. Folinic acid replenishes intracellular stores of reduced folic acid by competitively inhibiting cellular uptake of methotrexate. Methotrexate excretion can be enhanced by hydration with >3 L/m^2^ per day to maximize urine output [3]. Methotrexate can precipitate in the acidic urine causing crystalluria, hence alkalinizing the urine with oral or parenteral sodium bicarbonate can help prevent this and improve methotrexate excretion [18]. Another way to enhance elimination is via extracorporeal methods such as hemodialysis, high-flux hemodialysis, plasma exchange, and continuous renal replacement therapy [19]. In 2012, the US FDA approved glucarpidase for intravenous use in methotrexate toxicity in renal impairment. It is a recombinant form of carboxypeptidase G2 which is a bacterial enzyme that cleaves methotrexate into two catabolites. These catabolites are much less toxic than the parent compound and are excreted hepatically [20]. At present, data regarding the use of glucarpidase in low-dose methotrexate toxicity is lacking.

## 4. Conclusion

To conclude, it cannot be emphasized enough that methotrexate, although a very useful drug in the treatment of inflammatory arthritis, even when used in the lowest dose, may prove to be fatal in the presence of renal insufficiency. Routine CBC, liver, and renal function tests for patients on methotrexate should be performed at more frequent intervals in presence of chronic kidney disease. Myelosuppression from methotrexate can occur abruptly at any time during treatment. Methotrexate drug levels may not correspond with the severity of toxicity.

## Figures and Tables

**Table 1:** Studies, case series, and case reports regarding low-dose methotrexate toxicity.

Author	Year	Study type	Results
Nisar et al. [14]	1995	Retrospective study *N*=85	1 patient developed pancytopenia and died
Ohosone et al. [15]	1997	Prospective study *N*=284	Liver dysfunction: 3.2% Pancytopenia: 1.4% No fatality
Calvo-Romero et al. [12]	2001	Case series *N*=2	Pancytopenia and renal impairment in both cases with 1 death
Kivity et al. [10]	2014	Retrospective study *N*=28	Pancytopenia: 78.5% Hepatic dysfunction: 28%
Jariwala et al. [16]	2014	Case series *N*=2	Pancytopenia, renal dysfunction, and fatality in both cases
Mori et al. [13]	2016	Retrospective study *N*=40 (only patients with myelosuppression were included)	Myelosuppression can occur abruptly at any time during therapy
Mameli et al. [17]	2017	Case report	Pancytopenia, renal dysfunction, and death
